# The gene expression level of IFN-γR1 and IFN-γR2 in a murine model treated with *Toxoplasma* *gondii* and its products 

**Published:** 2016

**Authors:** Hamed Kalani, Hosein Khanahmad Shahreza, Ahmad Daryani, Hosein Ali Yousefi, Nader Pestechian, Vahid Mansouri

**Affiliations:** 1 *Department of Parasitology and Mycology, School of Medicine, Isfahan University of Medical Sciences, Isfahan, Iran*; 2 *Department of Genetics, School of Medicine, Isfahan University of Medical Sciences, Isfahan, Iran*; 3 *Toxoplasmosis Research Center, Mazandaran University of Medical Sciences, Sari, Iran*; 4 *Proteumics Research Center, Shahid Beheshti University of Medical Sciences, Tehran, Iran *

**Keywords:** *Toxoplasma gondii*, gene expression, IFN-γR1, IFN-γR2

## Abstract

**Aim:**

To evaluate the effect of active *T. gondii* tachyzoites and its products on the gene expression level of IFN-γR1 and IFN-γR2 in a murine model.

**Background:**

Many studies have shown that the parasite *Toxoplasma gondii* utilizes different mechanisms to inhibit the function of IFN-γ, but the parasite effect on the function of IFN-γR1 and IFN-γR2 is still unclear.

**Patients and methods:**

*Toxoplasma* lysate product (TLP), excretory/secretory products (ESPs) obtained from cell free and cell culture media as well as active tachyzoites were injected separately to their respective group each consisted of 10 BALB/c mice. One control group of 10 mice received phosphate buffered saline (PBS). All of the mice were euthanized three days after the last injection and then their peritoneal leukocytes were harvested separately. The total RNA was extracted from the samples, converted to cDNA, and the gene expression level of IFN-γR1 and IFN-γR2 was assessed in all of the treated groups relative to the control one.

**Results:**

There was no significant difference between each of the treated groups relative to the control group concerning the gene expression level of IFN-γR2 (P> 0.05). Furthermore, the gene expression level of IFN-γR1 in two groups of TLP (P= 0.04) and ESP obtained from cell free medium (P= 0.008) showed a significant difference relative to the control group.

**Conclusion:**

Findings of this study revealed a new aspect of host-*T. gondii* interaction in that this parasite is able to downregulate IFN-γR1 to reduce the IFN-γ effects on the infected cell.

## Introduction

*Toxoplasma *(*T*.) *gondii *is a parasitic protozoan with a worldwide prevalence (1). The adaptation of this parasite to numerous hosts makes it more difficult to control the disease caused by this parasite (1, 2). One reason for lack of success in controlling this parasite is that it utilizes various mechanisms to invade and proliferate in the host cell (3). The parasite entry into the host cell is completed less than 30 seconds and within the infected cell, *T*. *gondii *excretory/secretory products act as key factors in the survival and proliferation of this parasite (4). Although many aspects of host-*T*. *gondii* interaction has been studied by researchers, our knowledge in this field is still limited. For example, in spite of the fact that *T*. *gondii *genome harbors more than 8000 protein-encoding genes, the role of a few of these proteins has been revealed in the regulation of gene expression (5, 6). Furthermore, it has been proven that *T*. *gondii* is a great reservoir of microRNAs (miRNA), but the role of most of them in the regulation of gene expression is still unclear (7). Some studies have shown that the host cell gene expression regulatory factors such as NF-κB, SRF, MyD88 and c-Myc are manipulated by *T*. *gondii* parasite (3, 8). The most important cytokine with anti-toxoplasmic effect is IFN-γ (9). Several mechanisms induced by IFN-γ such as tryptophan starvation, increased nitric oxide (NO) production, and iron depletion are involved in the control of *T. gondii* infection (10). Therefore, all aspects of host- *T*. *gondii *interaction deserves to be realized as key points in the treatment and vaccination field against *T*. *gondii*.

Since no study has been performed to clarify whether any changes in the expression of IFN-γR1 and IFN-γR2 genes may be affected by *T*. *gondii* parasite or its derivatives *in vivo*, this study was aimed to assess whether or not active *T*. *gondii* tachyzoites and its products are able to alter the gene expression level of IFN-γR1 and IFN-γR2 in murine leukocytes *in vivo*. 

## Patients and Methods

***Parasite***

*T. gondii* tachyzoite, genotype І, strain RH, provided from the Toxoplasmosis Research Center of Mazandaran University of Medical Science, was maintained in the laboratory conditions through repeated passages in the peritoneal cavity of laboratory mice as well as cryopreservation (11). Since this parasite is highly virulent, so the use of it was according to the good laboratory practice (GLP) guideline. 

***Mouse***

Two mice strains, outbred Swiss Webster and inbred BALB/c mice, were used in this study. The former were used for the parasite maintenance in the laboratory conditions and the latter were used for the experiment. All of the BALB/c mice were female, 8 weeks old, and 20-25 grams. The University Research Ethics Committee (UREC) of the Isfahan University of Medical Science approved the use of the mice in this study (No. 191136). 

***T. gondii lysate product (TLP)***

TLP was prepared by lysing of tachyzoites. A high yield of tachyzoites was obtained from the ascitic fluid of intraperitoneally infected mice. For this purpose, the ascitic fluid of the infected mice was aspirated, centrifuged at 1500 ×g, 4 °C for 10 min and their supernatant was discarded. The tachyzoites obtained from each mouse were re-suspended in 2 ml of RPMI 1640 medium (Gibco Inc.), pooled, and washed three times washed with the same medium using centrifugation. After final washing, 25 ml of RPMI 1640 medium with 100 IU/ml of penicillin and 100 µg/ml of streptomycin (Sigma Inc.) was added to the precipitated tachyzoites in each 50 ml centrifuge tube. Tachyzoites were lysed using sonication in an ultrasonic bath filled with cold water (2-4°C) at 25 kHz, 30 s on and 10 s off for 5 min. The tubes were then centrifuged at 15000 ×g, 4°C, for 15 minutes and their supernatant was harvested, pooled, sterile filtered with 0.22-µm pore size filters (Denville Inc.) and stored as TLP at -20°C until use. To prevent alteration, no protease inhibitor was added to this product. 

***Excretory/secretory product (ESP) from cell*** ***culture medium***

ESP from cell culture medium was prepared from the culture of total murine peritoneal leukocytes. For this objective, about 2.4 × 10^8^ of the total peritoneal leukocytes was obtained from healthy Swiss Webster mice by washing the mice peritoneal cavity with RPMI 1640 medium. The resultant fluids were pooled and washed three times with RPMI 1640 medium using centrifugation at 1500 ×g, 4°C, for 10 minutes. The total leukocyte pellet was re-suspended in RPMI 1640 medium with 100 IU/ml of penicillin and 100 µg/ml of streptomycin. The number of leukocytes was adjusted to 2 × 10^6^ cells per ml of RPMI medium. Afterwards, 2 ml of the cell suspension was poured immediately into each well of 24-well cell culture plates (Sigma Inc.). The active tachyzoites, specified by the trypan blue exclusion test, were added to the wells at a ratio of 1:2 (leukocyte:parassite). The plates were then incubated at 37 °C, 5% CO_2,_ and 95% humidity for 48 h. Then, the supernatants of the wells were harvested, pooled in 50 ml centrifuge tubes on ice, centrifuged at 15000 ×g, 4 °C, for 15 minutes, and the supernatants of the tubes were harvested again, sterile filtered with 0.22-µm pore size filters and stored as ESP from cell culture medium at -20 °C until use. To prevent alternation, no protease inhibitor was added to this product. In addition, no serum (i.e. fetal bovine serum) was used in the cell culture medium in view of that it is rich in protein and makes this product of a low quality.

***ESP from cell free medium***

About 1 × 10^9^ active tachyzoites, obtained from intraperitoneally infected mice, were used to prepare ESP from cell free medium. For this purpose, tachyzoites were washed three times with RPMI 1640 medium using centrifugation at 1500 ×g, 4°C, for 10 minutes, and re-suspended in RPMI 1640 medium with 100 IU/ml of penicillin and 100 µg/ml of streptomycin. Subsequently, the parasitic suspension was divided into parts of 2 ml each containing 6 × 10^6^ tachyzoites in a centrifuge tube. The tubes were incubated under mild shaking at 37°C for 3 h and were centrifuged at 15000 ×g, 4°C, for 15 minutes. Their supernatant was harvested, pooled, sterile filtered with 0.22-µm pore size filters and stored as ESP from cell free medium at -20°C until use (12). No protease inhibitor was added to this product to prevent alteration. 

***Lyophilization***

The prepared *T. gondii* products were concentrated by lyophilization in the presence of trehalose (Sigma Inc.) (13, 14). Before lyophilization, trehalose was added to each product at a ratio of 10% (w/v). Subsequently, the products were lyophilized using a lyophilizer and kept at -20 °C until use. 

***Protein concentration of the lyophilized products***

The lyophilized products were reconstituted by adding 2 ml of phosphate buffered saline (PBS; pH: 7.4) to each of them. The concentration of protein was then calculated according to the method described by Bradford (15). 

***Injection to mice***

Injections were carried out intraperitoneally in all groups. For this purpose, 50 BALB/c mice were divided into 5 groups. Four groups were considered as test groups, three of which received one of the *T. gondii *products, including ESP from cell culture medium, ESP from cell free medium and TLP at 100-1000 µg doses for 1-10 mice, respectively, depending on their protein concentration. The fourth group received 1000-10000 active tachyzoites for 1-10 mice, respectively. Moreover, one group considered as a control group, received PBS at doses of 100-1000 µl for 1-10 mice, respectively. Exclusive of the active tachyzoite-receiving mice, the injections in the other groups were performed three times at 7-day intervals. The injection was performed in the active tachyzoite-receiving mice only once and three days before samples collection.

***Sample collection***

The peritoneal leukocytes, considered as sample, were collected from the mice in all groups. For this purpose, the mice were euthanized three days after the last injection and their peritoneal cavity was washed with RPMI 1640 medium. The harvested fluids from the mice were centrifuged separately at 1500 ×g, 4 °C, for 10 minutes, their supernatant was discarded, and immediately 1 ml of RNAlater^®^ solution (Qiagen Inc.) was added to each sample. The samples were stored at -20°C until use. 

***Total RNA extraction***

The total RNA was extracted from the collected samples using the Total RNA Purification Kit (Jena Bioscience Inc.), according to manufacturer’s instructions. To eliminate genomic DNA from the samples, on-column digestion was carried out using RNase-Free DNase Set kit (Qiagen Inc.). Subsequently, purity and concentration of the total RNA in samples were evaluated using NanoDrop^®^ ND-1000 spectrophotometer. The extracted total RNAs were then kept at -20°C until use.

***Reverse transcription-PCR (RT-PCR)***

The extracted total RNAs were converted to complementary DNA (cDNA) by the RT-PCR method. This was performed using AccuPower^®^ CycleScript RT PreMix (dN6) kit (Bioneer Inc.) according to manufacturer’s instructions. This procedure was performed as follows: the random hexamer primers were annealed at 15°C for 1 minutes and followed by cDNA was synthesized at 45 °C for 4 minutes. The enzyme reverse transcriptase (RT) was heat-inactivated at 95°C for 5 minutes. 

***Primer design***

The mRNA sequences of two target genes, including mouse IFN-γR1 and IFN-γR2 on chromosomes 10 and 16, respectively, as well as the reference gene mRNA sequence, including mouse hydroxymethylbilane synthase (HMBS) on chromosome 9 were extracted from the GeneBank^®^ home. Specific forward and reverse primers were designed for these genes using Beacon Designer™ software according to the SYBR^®^ Green method, considering that at least one primer spanned an exon-exon junction. More details concerning the designed primers have been presented in Table 1.

***Quantitative real time-PCR (Q-PCR)***

The gene expression level of IFN-γR1 and IFN-γR2 was evaluated by Q-PCR technique using Applied Biosystems StepOne™ Real-Time PCR System. This experiment was performed by qPCR GreenMaster with UNG kit (Jena Bioscience Inc.), according to manufacturer’s instructions. The reactions were carried out as follows: initial denaturation and polymerase activation was performed at 95°C for 2 minutes, and next 40 cycles of denaturation at 95°C for 15 s, annealing-extension at 60.2°C for 45 s. 

***Data analysis***

The normal distribution of data was analyzed by Kolmogorov–Smirnov (K–S) statistical test. The melting curve of Q-PCR products was examined for accuracy of the data. The gene expression levels of IFN-γR1 and IFN-γR2 in all of the test groups were assessed using the REST-2009 software (Qiagen, Inc.) relative to the control group. Moreover, this software was used to compare each test groups separately with the control one using *t*-test statistical analysis. In addition, the standard error of mean (SEM) for IFN-γR1 and IFN-γR2 ∆Ct was calculated for all groups.

**Table 1:** Details of the primer sequences designed in this study

Slope: Efficiency	Sequence	Primer	Accession number	Gene
-3.368: 0.976	CCGAGCCAAGGACCAGGATA	Forward	NM_013551.2^a^ NM_001110251.1^b^	HMBS
	TCAGGTACAGTTGCCCATCTTTC	Reverse		
-3.342: 0.990	CCTAAGTCCTTGCTCTCTGTGGTA	Forward	NM_010511.2	IFN-γR1
	TTCTTCCTGTTCTGCTGCTTCG	Reverse		
-3.361: 0.981	TCCTCGCCAGACTCGTTT	Forward	NM_008338.3	IFN-γR2
	GCCGCCTCCTGTTAAGTCA	Reverse		

a,b HMBS has two transcript variants, the homology of which was determined by MEGA4 software before primer design

## Results

The slopes and efficiencies obtained for the designed primers have been shown in Table 1. The gene expression level of IFN-γR1 was statistically significant between the TLP group and the control one (P= 0.04). 

**Table 2:** P-values of the relative gene expression level of IFN-γR1 and IFN-γR2 in the groups under study

P-values^1^				
	TLP	ESP-CF	ESP-CC	AT
IFN-γR1	0.04*	0.008*	0.32	0.13
IFN-γR2	0.8	0.73	0.66	0.84

1 The numbers with an asterisk (*) are significant statistically relative to the control group. TLP, *Toxoplasma gondii* lysate products; ESP-CF, excretory/secretory products from cell free medium; ESP-CC, excretory/secretory products from cell culture medium; AT, active tachyzoites

**Table 3:** The standard error of mean (SEM) for IFN-γR1 and IFN-γR2 in all of the groups

				Average ± SEM^a^							
TLP		ESP-CF			ESP-CC		AT			PBS	
IFN-R1	IFN-R2	IFN-γR1	IFN-R2		IFN-R1	IFN-R2	IFN-γR1		IFN-R2	IFN-R1	IFN-R2
3.85±0.9	4.3±1.35	4.33±0.73	5.12±1.41		2.48±0.65	4.9±0.35	2.62±0.33		4.43±0.44	1.65±0.5	4.58±0.6

a SEM was calculated for IFN-γR1 and IFN-γR2 ∆Ct in each of the groups. TLP, *Toxoplasma gondii* lysate products; ESP-CF, excretory/secretory products from cell free medium; ESP-CC, excretory/secretory products from cell culture medium; AT, active tachyzoite; PBS, phosphate buffered saline

**Figure 1:**
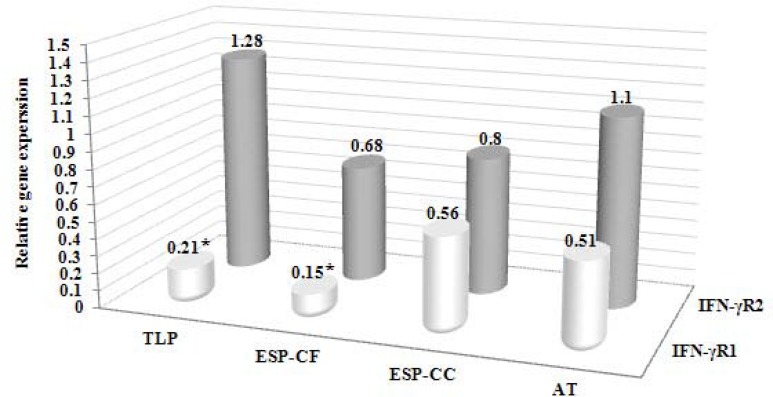
The relative gene expression level of IFN-γR1 and IFN-γR2 in the groups under study. The gene expression level of the columns marked with an asterisk (*) are significant statistically relative to the control group (*P* < 0.05). TLP, *Toxoplasma gondii* lysate products; ESP-CF, excretory/secretory products from cell free medium; ESP-CC, excretory/secretory products from cell culture medium; AT, active tachyzoites

Additionally, there was a significant difference between the groups of the ESP from the cell free medium and the control (*P*= 0.008). Moreover, there was no significant difference among the test groups and the control regarding the expression level of IFN-γR2 gene. The expression level of IFN-γR1 gene in the TLP group and the group of the ESP from cell free medium was 0.21 and 0.15 relative to the control group, respectively. The obtained *P*-values for the relative gene expression level of two target genes, IFN-γR1 and IFN-γR2, in all test groups has been shown in Table 2. Furthermore, the relative gene expression values of IFN-γR1 and IFN-γR2 in the above-mentioned groups has been presented in Fig. 1. Additionally, the obtained SEM for all groups has been given in Table 3. 

## Discussion

Many studies have been conducted regarding the interaction between *T. gondii* and the host defensive responses. Several studies have shown the induction of the host immune responses is varied depending on the parasite genotype (13). One example in this matter is that genotype 2 strongly induces the production of IL-12 while genotype 1 has no such effect (14). The increased level of IL-12 leads to a high production of IFN-γ that causes the parasite to be encysted in host tissues (15), the reason why the number of *T. gondii* tissue cysts in genotype 2 is higher than that in genotype 1. Studies revealed that a reduction in the IFN-γ gene expression level is responsible for the egression of the parasite and turn it into the form called tachyzoite (16). Therefore, the last-mentioned molecule is essential for stage conversion of this parasite as well as keeping it in the cystic form (17). This parasite utilizes a wide variety of mechanisms to inhibit the IFN-γ effects on the infected cell. For example, ROP16, a rhoptry-related protein kinase, directly activates signal transducer and activator of transcription factor 3 (STAT3) in the infected cell (18) that can lead to the inhibition of apoptosis (19), the suppression of IL-12 production (20), and the induction of IL-10 production (21). Furthermore, ROP16 activates STAT6, resulting in the induction of IL-4 production (22, 23). Obviously, two mentioned cytokines, IL-10 and IL-4, inhibit the IFN-γ effects on the infected cell (24). Moreover, it has been revealed that another rhoptry protein kinase, ROP18, is capable of inhibiting the NF-κB signaling pathway in the infected cell, leading to the suppression of proinflamatory cytokines production such as IFN-γ (25). In addition, *T. gondii* increases the gene expression level of suppressor of cytokine signaling 1 (SOCS1) protein to limit JAK phosphorylation on the cytoplasmic tail of IFN-γR2, leading to the inhibition of IFN-γ-related signaling pathway into the infected cell (26). Dysregulation of IFN-γ-responsive genes is also another mechanism by which *T. gondii* block the IFN-γ effects on the infected cell (27). All of the above-mentioned mechanisms occur inside the infected cells with *T. gondii *parasite. Interestingly, it has been demonstrated that the serum level of IFN-γ increases in the infected host with *T. gondii *(28, 29). This occurs because of the fact that when the parasite is within the tissue cyst releases some of its excretory/secretory products into the host body (i.e. antigen shedding) and stimulates IFN-γ production. An increased level of IFN-γ holds the parasite in the cystic form (10) that is crucial for parasite survival in nature. Therefore, this parasite is able to selectively decrease or increase IFN-γ-relating functions inside or outside of the infected cell, respectively. Authors also showed that anti-toxoplasma activity of macrophages strongly depends on binding of IFN-γ to IFN-γR1 (9, 30). In the present study, no significant changes were observed in the gene expression level of IFN-γR2 in the groups under study. However, IFN-γR1 gene expression was decreased noticeably in the groups of TLP and ESP from cell free medium relative to the control one. The gene expression of IFN-γR1 in the groups of ESP from cell culture medium and active tachyzoite was evidently almost half of that in the control group. However, it was not statistically significant. It appears that the compound of ESP from cell culture medium is as similar as that of released by the active parasite *in vivo*. Therefore, the similar effect of active parasite and ESP from cell culture medium regarding the gene expression level of IFN-γR1 is justifiable. According to what was discussed above, *T. gondii* inhibits IFN-γ effects on the infected cell through several mechanisms as follows: the inhibition of IFN-γR2-related signaling pathway (26) (but no alteration in IFN-γR2 gene expression as shown in the present study), the increased level of IL-4 and IL-10 production (20-23), the inhibition of IFN-γ-inducible genes (27), and IFN-γR1 downregulation (as shown in the present study). 

Findings of this study revealed a new aspect of host-*T. gondii* interaction in that this parasite is able to downregulate IFN-γR1 to reduce the IFN-γ effects on the infected cell.

## Note

**(**Please cite as:** Kalani** **H, Khanahmad Shahreza** **H, Daryani** **A, Yousefi** **HA, Pestechian N, Mansouri** **V. The gene expression level of IFN-γR1 and IFN-γR2 in murine model treated with *****Toxoplasma gondii***** and its products. Gastroenterol Hepatol ****Bed Bench**** 2016;9(2):124-131).**
